# Metabolic dysfunction associated steatotic liver disease—Clinicians should not underestimate the role of steatosis

**DOI:** 10.1002/ueg2.12520

**Published:** 2024-01-04

**Authors:** Johannes Wiegand, David Petroff, Thomas Karlas

**Affiliations:** ^1^ Division of Hepatology Department of Medicine II Leipzig University Medical Centre Leipzig Germany; ^2^ Clinical Trial Centre Leipzig University of Leipzig Leipzig Germany; ^3^ Division of Gastroenterology Department of Medicine II Leipzig University Medical Centre Leipzig Germany

**Keywords:** controlled attenuation parameter (CAP), FAST score, fibrosis, transient elastography

All‐cause mortality and incidence of liver related complications of metabolic dysfunction associated fatty liver disease (MASLD), formerly named nonalcoholic fatty liver disease (NAFLD), are mainly driven by the extent of liver fibrosis.^1^ Hepatic steatosis has been considered less relevant, however, recent data have shown that hepatic fat accumulation deserves more attention: (i) individuals with grade 3 steatosis show higher overall mortality and development of type 2 diabetes mellitus (T2DM) than cases with less steatosis. Reduction of hepatic fat decreases the risk of developing T2DM,^2^ (ii) hepatic steatosis increases the risk for hepatocellular carcinoma,^3^ and (iii), hepatic steatosis measured by means of elevated controlled attenuation parameter (CAP) values (>238 dB/m) in patients with T2DM correlates with a composite endpoint driven by cerebrovascular insult and chronic kidney disease.^4^ Due to these observations and the epidemiological burden of MASLD, there is the clinical need to quantify steatosis in daily routine with the following prerequisites:

‐ easy to obtain and noninvasive
‐ reliable, reproducible, and prospectively applicable
‐ affordable

Controlled attenuation parameter is a quantitative ultrasound based technique to noninvasively characterize hepatic steatosis. It is an add‐on software to the fibroscan® device—the standard of care for ultrasound based noninvasive fibrosis characterization of MASLD in clinical guidelines ‐ and quantifies attenuation of the ultrasound tracking impulses during liver stiffness measurement and delivers the attenuation slope with a range of 100–400 dB/m.^5^, ^6^, ^7^ CAP fulfills most of the aforementioned criteria, however, only about 140 fibroscan^®^ devices are currently available in Germany, for example, and these are mainly restricted to secondary and tertiary care.^8^, ^9^, ^10^ Moreover, they are not universally equipped with the CAP software, which would be quite expensive for a stand‐alone tool for quantification of steatosis. This is mitigated by the fact that the parameters liver stiffness, CAP value, and aspartate aminotransferase (AST) can be combined to the FAST score as estimation of fibrotic steatohepatitis.^11^

In the present issue of UEG journal, Bianco and colleagues developed the steatosis score “CAPS” to predict CAP values ≥275 dB/m, which was taken as a surrogate for hepatic fat.^12^ It is based on the readily available clinical parameters age, BMI, abdominal circumference, HbA1c, ALT and HDL and was derived in a cohort of blood donors with at least three items of metabolic dysfunction (overweight, hypertension, fasting glucose/diabetes/HbA1c, low HDL, or increased triglycerides). The CAPS score was validated in three additional cohorts including the general population and individuals scheduled for bariatric surgery. The AUROC to predict a CAP value ≥275 dB/m was 0.73. The authors conclude that the CAPS score is useful to identify MASLD in high‐risk individuals.

Investigation of the CAPS score in four different cohorts spanning a broad spectrum where MASLD is clinically important is a strength of the Bianco paper. However, the accuracy of the score is only moderate, a control with liver histology or magnetic resonance based proton density fat fraction (MR‐PDFF) is missing, and prospective longitudinal data or correlation with clinical endpoints do not yet exist. The use of “healthy”, middle aged blood donors for the derivation cohort led to a predominantly male (83%) population with almost no diabetes (1.5%). Furthermore, the CAPS score is not meaningfully better in performance than the established Fatty Liver Index in the validation cohorts, although the abstract implied the contrary by stating results from the derivation cohort. Nevertheless, it is important that the authors focus on the clinical relevance of steatosis in the disease continuum from MASLD and MASH to advanced stages of fatty liver disease. The community has to develop adequate noninvasive tools to screen for MASLD and longitudinally monitor treatment effects of upcoming therapeutic options like the thyroid receptor beta agonist resmetirom which has recently successfully passed phase 3 development.^13^ These tools finally should also predict hepatic and cardiovascular endpoints to identify patients suitable for pharmacological interventions and define a population that is not at risk and does not unnecessarily tie up scarce health care resources.
